# Inutility and Dangers of Medical Treatment of Epithelioma of the Tongue

**Published:** 1881-09-20

**Authors:** 


### INUTILITY AND DANGERS OF MEDICAL TREATMENT OF EPITHELIOMA OF THE TONGUE.

Scattered throughout the Bulletins de la Societe de Chirurgie, for the year 1879, are found the reports of a discussion which fully brought out the opinion of M. Verneuil, perhaps the most eminent surgeon of the present French Faculty, regarding the folly of temporizing with cancer of the tongue.

The conclusions he formulated are the following:

1. Topical treatment cannot cure true lingual cancroid. Mercury has no action except on syphilitic symptoms which were formerly difficult of recognition, but which, through the works of Fournier, have become of facile diagnosis. Iodide of potash has no real efficiency against true neoplasm; its use should be restricted to syphilitic and scrofulous lesions. Finally, chlorate of potash sometimes cures ulcerations and adenomata of the sudoriparous glands, (ulcerations et adenomes sudoripares) but otherwise merits no confidence.

2. All these medicaments, as also the milder caustics, often so deplorably insisted on, are not only inefficacious, but even productive of injury. But there is no medicament which precipitates the onward march of cancer like mercury, which again, like iodide of potash, often induces grave perturbations in the digestive functions.

3. At the debut there is always a moment when the malady is sufficiently characterized and its nature demonstrated, while it is yet so circumscribed that ablation may be performed without fear of complication.

4. But why operate, say many, when relapse (recidive) will surely occur? When ablation is done before the period of propagation and cancerous cachexia there is always hope of definite cure.

M. Verneuil cites four of his patients who were completely cured. The epithelioma was but about the length of the nail, and had little depth in three of the cases. The fourth patient had been operated on previously, and there was one ganglion affected; nevertheless, after the operation, the morbid tissue was not reproduced.—*Med. and Surg. Reporter.*

